# The complete mitogenome of *Pareuchiloglanis sichuanensis* (Siluriformes: Sisoridae)

**DOI:** 10.1080/23802359.2021.1975504

**Published:** 2021-09-19

**Authors:** Taiming Yan, Qian Zhang, Jiayang He, Feiyang Yan, Zhijun Ma, Hongjun Chen, Lijuan Ye, Nan Zhang, Deying Yang, Zhi He

**Affiliations:** College of Animal Science and Technology, Sichuan Agricultural University, Chengdu, China

**Keywords:** *Pareuchiloglanis sichuanensis*, mitochondrial genome, phylogenetic analyses

## Abstract

*Pareuchiloglanis sichuanensis* is an endemic fish species in the upper reaches of the Yangtze River. In the present study, the complete mitochondrial genome of *P. sichuanensis* was analyzed. The mitochondrial genome, consisting of 16,774 base pairs (bp), included 13 protein-coding genes, 2 ribosomal RNAs, 22 transfer RNAs, and a non-coding control region. The phylogenetic tree showed that *P. sichuanensis* was closely related to *P. anteanalis*. These results provid the useful information for further studies on taxonomic status, molecular systematics, and stock evaluation.

*Pareuchiloglanis sichuanensis* Ding, Fu, Ye 1991 is categorized into family Sisoridae, order Siluriformes (Ding et al. 1991), distributed in the Dadu River, Qingyi River, and Min River, which all belong to the upper reaches of the Yangtze River drainage (Li et al. 2020). It usually lives in the bottom of streams and rivers, where the bottom material is sand and stone (Ding et al. 1991). The fish populations have declined dramatically in recent years as a result of over fishing, dam construction, water pollution, and other human interferences. However, the studies on this species were only limited in some reports about the taxonomic characters and distribution (Ding et al. 1991; Li et al. 2020). Therefore, some basic biology data including genetic information should be further studied, which may be beneficial to research on systematics, resource protection and development of *P. sichuanensis*. In this study, we sequenced, assembled, and annotated the complete mitochondrial genome of *P. sichuanensis*, which could provide the useful genomic resources for the future studies.

In the present study, the *P. sichuanensis* specimens were collected from the upstream of Tianquan River, a tributary of the Qingyi River (N: 29°57′17.0252251200″, E: 102°26′5 .3818130400″) and chosen for mitochondrial genome analysis in 2020. The voucher samples were deposited at Aquaculture Department of Sichuan Agricultural University (Zhi He, zhihe@sicau.edu.cn, under the voucher number psi202004). Then, the total genomic DNA was extracted from the muscle by a traditional phenol-chloroform method. Whole genome sequencing was conducted with Illumina Novaseq platform. The mitochondrial genome was assembled de novo using A5-miseq(v20150522) (Coil et al. 2015) and SPAdes (v3.9.0) (Bankevich et al. 2012), and then annotated using the MITOS Webserver (Bernt et al. 2013).

The complete mitogenome of *P. sichuanensis* was a circular molecule with a length of 16,774 bp, consisting of 13 protein-coding genes, 2 ribosomal RNAs, 22 transfer RNAs, and a non-coding control region. The nucleotide composition of *P. sichuanensis* genome was A 31.69%, T 25.47%, G 15.33%, and C 27.50%, with a high A + T content of 57.16%. The *nad6* and eight tRNA genes (*tRNA-Gln*, *tRNA-Aln*, *tRNA-Asn*, *tRNA-Lys*, *tRNA-Tyr*, *tRNA-SerUCN*, *tRNA-Glu*, and *tRNA-Pro*) were encoded on the light-strand. On the contrary, all the other genes were encoded on the heavy-strand. This was a typical gene arrangement conforming to the other *Pareuchiloglanis* species and vertebrate consensus (Cui et al. 2019). The genome sequence data that support the findings of this study is openly available in GenBank of NCBI at (https://www.ncbi.nlm.nih.gov/) under the accession no. MW697900.

To better understand the phylogenetic relationships of mitochondrial sequences in *Pareuchiloglanis*, we selected seven *Pareuchiloglanis* species (Figure 1). Based on the concatenated amino acid sequences of 13 proteins, the phylogenetic tree was constructed using the Maximum Likelihood method (Jones et al. 1992; Kumar et al. 2016) (Figure 1). The results of phylogenetic analysis indicate that all *Pareuchiloglanis* species have the close relationship, and *P. sichuanensis* and *P. anteanalis* are monophyletic in the tree. Thus, the mitochondrial genome data and phylogenetic analysis of the *P. sichuanensis* enrich the evolution research of *Pareuchiloglanis*.

**Figure 1.**
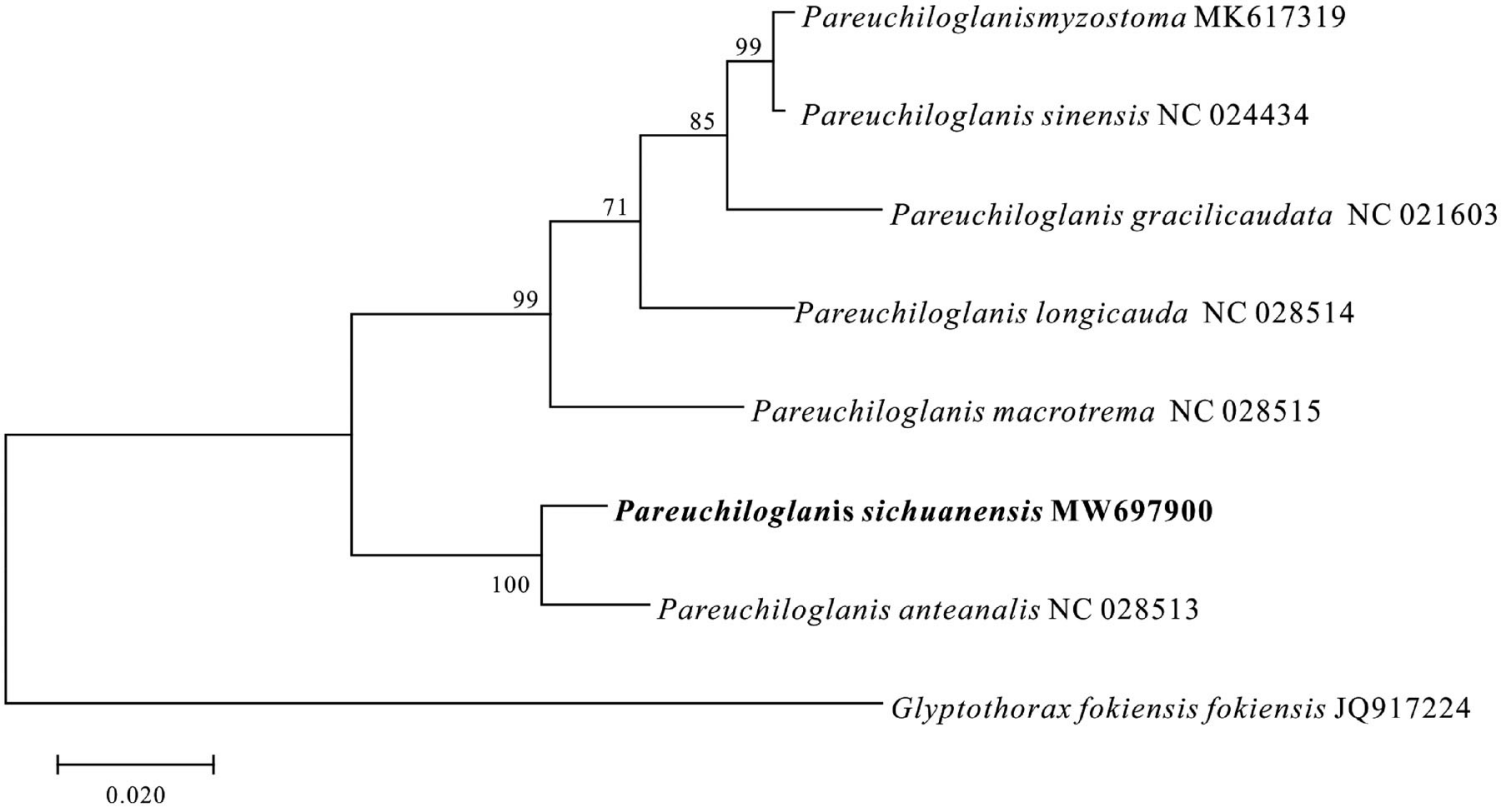
Molecular phylogenetic analysis by Maximum Likelihood method for eight *Pareuchiloglanis* species was inferred from concatenated amino acid sequences data of 13 mitochondrial proteins. Node labels indicate the bootstrap values.

## Data Availability

The genome sequence data that support the findings of this study is openly available in GenBank database under the accession number MW697900 (https://www.ncbi.nlm.nih.gov/nuccore/MW697900). The associated BioProject and Bio-Sample numbers are PRJNA752914, SAMN20667725, and SAMN20667726, SRA for short reads and long reads are SRR15374433 and SRR15374432, respectively.
